# Taxing working memory during laparoscopic training: reducing the impact of perfectionistic concerns on skill learning

**DOI:** 10.1007/s00464-026-12739-z

**Published:** 2026-03-25

**Authors:** V. E. E. Kleinrensink, L. W. Kranenburg, G. J. Kleinrensink

**Affiliations:** 1 Department of Neuroscience, Erasmus University Medical Center, Dr. Molewaterplein 40, 3015 GD Rotterdam, The Netherlands; 2 Department of Psychiatry, Erasmus University Medical Center, Dr. Molewaterplein 40, 3015 GD Rotterdam, The Netherlands

**Keywords:** Minimally invasive surgery, Laparoscopic training, Perfectionism, Working memory, Simulation, Skill retention

## Abstract

**Background:**

Perfectionistic concerns are common among surgeons and have been linked to reduced learning gains in the acquisition of basic instrument-handling skills for minimally invasive surgery (MIS). These concerns often trigger rumination, which distracts attention from the task at hand during practice and interferes with effective skill acquisition. According to working-memory models, occupying task-irrelevant capacity with a neutral secondary task may leave fewer resources available for rumination, thereby reducing negative self-focus. This study investigated whether adding a secondary working-memory task during MIS training alters the association between self-critical perfectionism and laparoscopic skill retention.

**Methods:**

In this randomized experimental study, 55 laparoscopically naïve participants practiced a peg-transfer task on a custom MIS simulator and returned within 48 h for a retention test. Participants were allocated to a control condition wherein the target position for the pegs were continuously visible, or an experimental condition in which targets were visible for one second only, imposing visuospatial working-memory load. Performance was measured by completion time and instrument path length. Perfectionistic strivings and perfectionistic concerns were measured with the Big Three Perfectionism Scale. Retention was measured as the regression slope between late-practice and retention performance, wherein more negative slopes reflect better retention.

**Results:**

Practice performance and retention test performance did not differ between conditions. Perfectionistic strivings were not associated with skill retention in either group. In the control condition, higher perfectionistic concerns significantly predicted reduced retention of movement efficiency, though completion time was unaffected. Conversely, under working-memory load, perfectionistic concerns did not predict retention for either outcome.

**Conclusions:**

Imposing a visuospatial working memory load during MIS practice did not compromise performance, and the observed pattern is consistent with reduced vulnerability to perfectionistic concerns in movement-efficiency retention. While these initial patterns are promising, future studies are required to definitively establish the effectiveness of these training constraints.

Patient safety in health care relies heavily on the ability of physicians to effectively identify and manage adverse events. Consequently, consistent self-evaluation and adherence to high standards are central aspects of medical professionalism [1–4]. However, when personal identity and self-worth become overly dependent on flawless performance, this healthy pursuit can turn into perfectionism [5]. Given the demanding nature of the field, it is perhaps not surprising that perfectionistic tendencies are common among surgeons [6, 7], surgical residents [8, 9], and medical students [10, 11]. In the context of medical training, this trait can significantly impede learning [12–14]. Specifically, in minimally invasive surgery (MIS), perfectionistic concerns have been associated with reduced learning gains [15]. Mastery of MIS skills requires a significant investment by the surgeon, yet available practice time may not always suffice to meet the training needs of surgical residents [16]. Therefore, to assist trainees in maximizing their use of available time, it is necessary to explore strategies to mitigate the impact of perfectionistic concerns during MIS skill practice.

Research has provided a framework for understanding the effects of perfectionism by defining it through two dimensions: perfectionistic strivings and perfectionistic concerns [17, 18]. Perfectionistic strivings involve setting high personal standards and actively engaging in problem solving to achieve these goals. Individuals with this trait often tie their self-worth to their achievements. As a result, they continually raise their standards when they achieve their goals [19]. While this drive can increase self-efficacy and promote goal attainment [20], in excess it may become an all-consuming preoccupation that can cause mental and physical harm [21]. In contrast, perfectionistic concerns are characterized by a fear of negative evaluation if standards are not met [22]. Individuals with high perfectionistic concerns focus intensely on perceived shortcomings, which can inhibit constructive problem solving [23]. This focus often leads to task avoidance and diminished motivation over time [24], generating additional tension as self-worth remains tied to performance based on rigid standards [25]. Moreover, even when these individuals achieve their goals, they typically derive little satisfaction, viewing success merely as meeting expected obligations rather than as a source of fulfillment [26]. This mindset is associated with anxiety, depression, and burnout [27–30].

A potential solution to mitigate the negative impact of perfectionistic concerns on learning, is to target the underlying cognitive processes. Perfectionistic concerns frequently give rise to rumination, where individuals fixate on others' evaluations and dwell on past mistakes [31]. These thoughts take up mental resources and can interfere with effective learning and task performance. According to Baddeley’s multi-component model of working memory, the brain’s central executive has limited capacity [32, 33]. This means that if the working memory space that is not required for the task itself is occupied with a neutral secondary task, there is less mental capacity available for rumination to occur. Previous studies have shown that such neutral cognitive load reliably distracts from negative thoughts and helps emotional recovery after errors [34].

Targeting working memory during task performance presents a promising strategy to reduce the harmful effects of perfectionism. For learners vulnerable to perfectionism, this may provide a stabilizing effect, allowing them to stay focused when making errors or experiencing performance anxiety during MIS practice. In addition, prior studies have demonstrated that multitask training during the acquisition of basic laparoscopic skills promotes more implicit and automatic motor control compared to single-task training [35, 36].

To our knowledge, no research has yet explored whether adding working memory load during practice can alleviate the negative impact of perfectionistic concerns on learning outcomes in laparoscopic skills. To address this gap, we tested a MIS training task in which reliance on visuospatial working memory was increased. We explored whether this training context was associated with a weaker relationship between perfectionistic concerns and retention outcomes compared with standard visually guided practice. We hypothesized that the association between perfectionistic concerns and retention would be weaker in the working memory load condition than in the control condition. We expected that increasing cognitive demands during practice might limit self critical rumination and thereby support learning for individuals reporting higher perfectionistic concerns.

## Materials and methods

### Participants

A total of 55 participants completed the study, of whom 27 were randomly assigned to the control condition and 28 to the experimental condition. The mean age of participants was 18.8 years (SD = 1.23), with 21 male and 34 female participants. All participants reported that they had no prior laparoscopic experience. Baseline characteristics did not significantly differ between groups.

### Apparatus

To assess MIS skill progression, a custom laparoscopic training setup was used. The system simulated minimally invasive surgery (MIS) by occluding direct vision of the instrument movement. A fixed camera captured the task area, and video images were displayed on a 17-inch monitor positioned at eye level. The Leap Motion Controller (Leap Motion Inc., Model LM-010) was used to track instrument movement. The device operates with three infrared LEDs and two CCD cameras, providing high-resolution motion tracking. It was placed horizontally in front of the task area to maintain constant visibility of the tracked instrument. A white rubber tube attached to the instrument’s tip was used for motion tracking. The tube was recognized as a pen-shaped object by the LMC for precise position measurements. A custom mount supported both the LMC and the camera. LMC driver software (version 2.3.1) tracked motion data, while custom software, developed in Python, extrapolated positional data to simulate instrument tip movements. OpenCV 2 was used to superimpose task figures onto the video feed and track the positions of colored objects. For more details regarding the apparatus, see [37].

### Task: experimental vs control condition

**Fig. 1:**
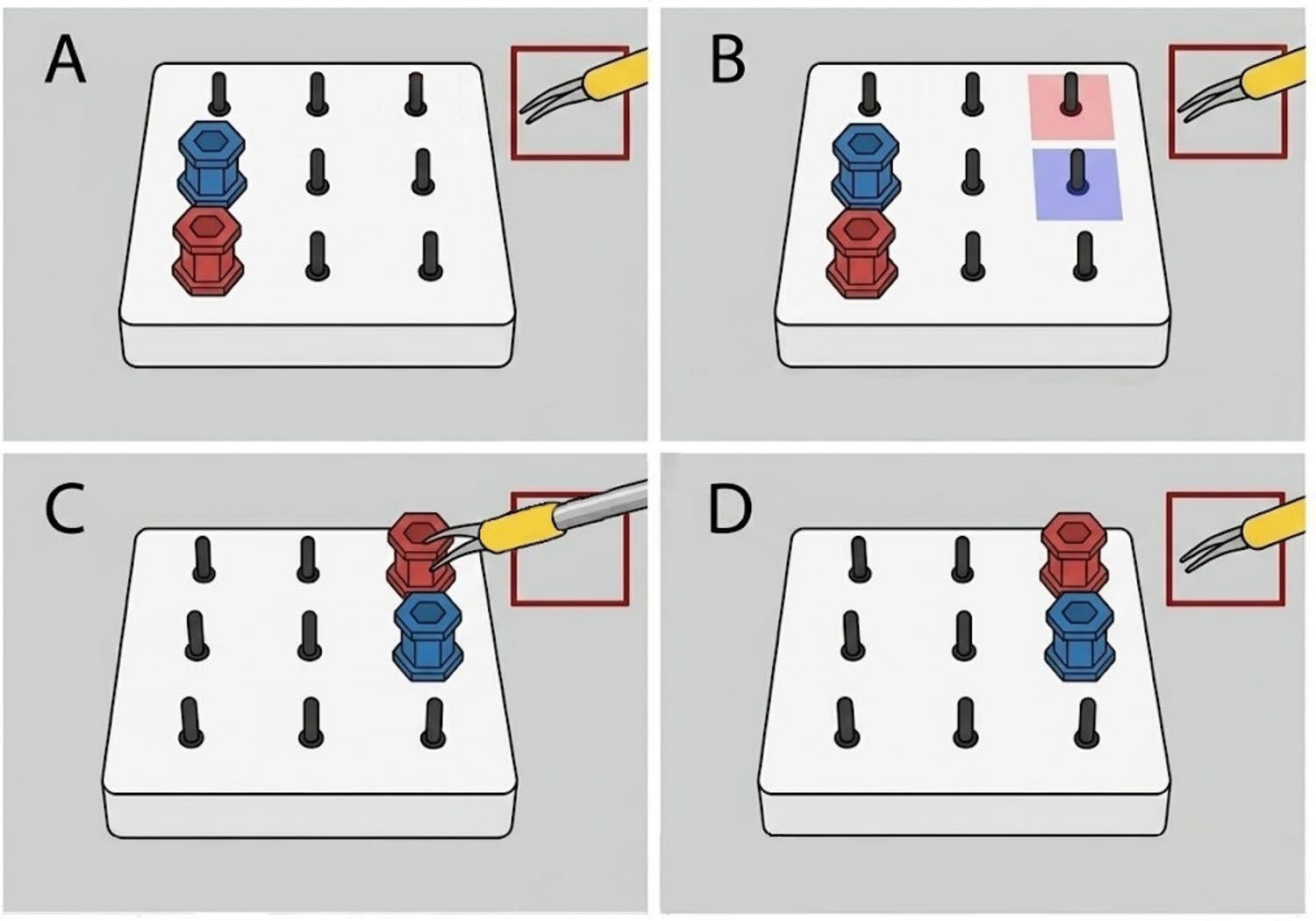
Step-by-step visual sequence of the peg transfer task: **A** Initiation of a trial, **B** Display of the target configuration, **C** Placement of pegs in the designated positions on the task board, **D** Completion of the trial and transition to the next one

A modified version of the peg transfer task, adapted from the Fundamentals of Laparoscopic Surgery (FLS) program [38], was used to assess laparoscopic skills. In this task, participants used a grasper to move two colored pegs across a board to designated target positions, displayed as colored squares on a monitor (Fig. 1). In the control condition, the target positions remained visible on the monitor throughout each trial.

In the experimental condition, we increased working memory demands by displaying the target positions for 1 s at the start of each trial and then removing them. Participants therefore had to retain the target locations in working memory while executing the task without online target cues. This approach is widely used in visuomotor research to elicit memory-guided actions and increase reliance on stored spatial representations [39].

For left-handed participants, the sequence of positions was mirrored. Participants used the dominant hand to perform the task. If a peg was dropped, they were instructed to pick it up manually and place it in the middle of the task board before continuing. Peg drops were not logged separately, because any drop required retrieval and re-grasping before continuing and was therefore expected to increase completion time and instrument path length.

### Measures

Performance on the surgical task was assessed per trial by total time to completion and total path length of the instrument. To determine the degree of perfectionist characteristics, the Big Three Multidimensional Perfectionism Scale (BTPS) was used. This questionnaire consists of 45 items and captures three higher-order dimensions of perfectionism: Rigid Perfectionism (self-oriented perfectionistic standards and self-worth contingencies), Self-Critical Perfectionism (concern over mistakes, doubts about actions, self-criticism, and socially prescribed perfectionism), and Narcissistic Perfectionism (other-oriented perfectionism, hypercriticism, entitlement, and grandiosity). Items are rated on a 5-point Likert scale ranging from 1 (strongly disagree) to 5 (strongly agree).

Subscale scores were calculated as the sum of item responses, with higher scores indicating higher levels of the trait. Rigid Perfectionism comprises 10 items (range 10–50) and Self-Critical Perfectionism comprises 17 items (range 17–85) [40].

The authors of the scale demonstrated strong reliability and construct validity for the BTPS [40]. For the purpose of this study, we administered two BTPS subscales: Rigid Perfectionism, which reflects *perfectionistic strivings* (high personal standards and achievement-oriented self-discipline), and Self-Critical Perfectionism, which reflects *perfectionistic concerns* (fear of mistakes and negative self-evaluation).

### Procedure

Upon arrival, participants were randomly allocated to either the control or experimental condition. After allocation, they provided written informed consent and completed the BTPS questionnaire. The experimenter then demonstrated the peg transfer task and provided standardized instructions on instrument handling.

Participants received no quantitative performance feedback during practice (i.e., no display or review of completion time or instrument path length). Feedback was limited to intrinsic visual information from the task. The experimenter provided standardized instructions only and did not provide coaching or evaluative comments.

Participants completed 40 consecutive practice trials on the surgical simulator under their assigned condition. Within 48 h, they returned for a retention session consisting of 10 additional trials using the same peg configurations as in the final practice block. To ensure that retention reflected only skill learning, independent of the earlier working-memory manipulation, the target positions were kept visible during all retention trials for both groups.

### Statistical analysis

Skill retention was assessed by calculating the slope of the regression line between average performance on the last ten trials of the practice session and the ten trials of the retention session. This gives a single retention score that reflects how much of each participant’s final practice level was maintained at retention, while accounting for the fact that participants may end practice at different performance levels. As lower values on both outcome measures reflect higher efficiency in task execution, negative slopes would imply increased performance retention, whereas positive slopes (or relatively less-negative slopes) would represent decreased retention.

To examine whether perfectionism was associated with retention, we first conducted simple linear regressions within each condition to estimate condition-specific associations between BTPS dimensions and retention slopes. To formally test whether these associations differed by condition, we fitted additional models that included the main effects of condition and perfectionism, alongside their interaction term. However, given our modest sample size and the continuous distribution of trait scores, we acknowledge that the study was likely underpowered to detect significant interaction effects. We used an alpha level of 0.05 for all statistical tests. All analyses were performed in SPSS 24.

## Results

### Questionnaires

Across all participants, mean Self-Critical Perfectionism was 40.95 (SD = 11.28) and mean Rigid Perfectionism was 25.00 (SD = 7.86). These subscale sum scores correspond to average item endorsements of 2.41 and 2.50, respectively. Scores did not differ between the control and experimental conditions for either Self-Critical (*t*(53) =  − 1.59, *p* = 0.118) or Rigid Perfectionism (*t*(53) =  − 0.58, *p* = 0.564).

### Practice session

At baseline, the working-memory load group had a mean completion time of 66.72 s (SD = 37.43) and a mean path length of 2391.23 mm (SD = 1724.96). The control group had a mean completion time of 62.35 s (SD = 40.67) and a mean path length of 2412.91 mm (SD = 2614.83). Neither Self-Critical Perfectionism nor Rigid Perfectionism predicted baseline completion time or baseline path length (all ps > 0.05).

Across all participants, the mean duration of the practice session was 18.31 min (SD = 5.45), and the total path length during practice averaged 37,145 mm (SD = 17,123).

Total practice duration did not differ between control and experimental conditions (*t*(53) = 0.576, *p* = 0.567), nor did total practice path length (*t*(53) = 0.800, *p* = 0.427).

During the last ten practice trials, neither Self-Critical Perfectionism nor Rigid Perfectionism predicted mean completion time or mean path length (all ps > 0.05), indicating that perfectionism scores were not associated with performance at the end of practice.

### Retention session

The mean retention completion time per trial was 19.82 s (SD = 3.97) in the control group and 20.90 s (SD = 7.04) in the experimental group, with no significant between-group difference (*t*(53) = 0.526, *p* = 0.601).

For path length per trial, the control group demonstrated a mean of 768 mm (SD = 300) and the experimental group 784 mm (SD = 433), again with no significant difference between groups (*t*(53) = 0.278, *p* = 0.782).

Retention of learning was quantified as the regression slope between the final ten practice trials and retention trials (more negative values indicate greater retention of efficiency).

The mean slope for completion time was 0.53 (SD = 7.54) in the control condition and − 0.84 (SD = 5.08) in the experimental condition, with no significant group difference (*t*(53) = 0.779, *p* = 0.440). For path length, the mean retention slope was 80.03 (SD = 432.43) in the control condition and 134.74 (SD = 345.20) in the experimental condition, again with no significant difference (*t*(53) =  − 0.515, *p* = 0.609).

### Perfectionism and retention

To examine whether perfectionism was associated with retention, we ran simple linear regressions separately within each condition.

In the control condition (no working-memory load), higher Self-Critical Perfectionism predicted poorer retention of movement efficiency, reflected by a higher path-length retention slope (*B* = 9.65, 95% CI [0.40, 18.90], *t*(25) = 2.15, *p* = 0.042, *R*^2^ = 0.156, *β* = 0.395). Self-Critical Perfectionism was not associated with retention of completion time (*B* =  − 0.039, 95% CI [− 0.250, 0.172], *t*(25) =  − 0.38, *p* = 0.705, *R*^2^ = 0.006, *β* =  − 0.076). Rigid Perfectionism was not associated with retention slopes for either path length (*B* = 11.30, 95% CI [− 2.05, 24.65], *t*(25) = 1.74, *p* = 0.093, *R*^2^ = 0.108, *β* = 0.329) or completion time (*B* = 0.037, 95% CI [− 0.260, 0.333], *t*(25) = 0.25, *p* = 0.801, *R*^2^ = 0.003, *β* = 0.051).

**Fig. 2:**
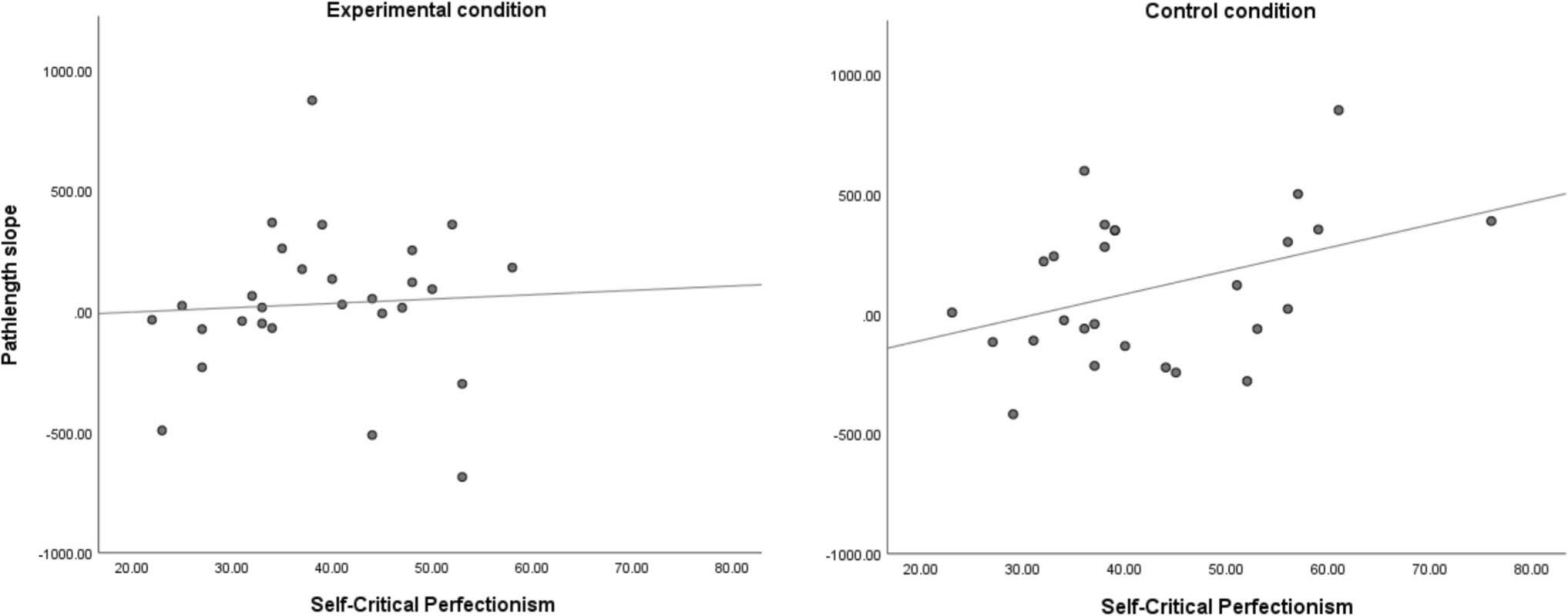
Scatterplots showing the association between Self-Critical Perfectionism (BTPS) and the path length retention slope in the working-memory load condition (left) and control condition (right). Positive slopes reflect poorer retention of movement efficiency, whereas more negative slopes reflect greater retention

In the working-memory load condition, neither perfectionism dimension was associated with retention. Self-Critical Perfectionism was not associated with path-length retention (*B* = 1.80, 95% CI [− 10.59, 14.20], *t*(26) = 0.30, *p* = 0.767, *R*^2^ = 0.003, *β* = 0.059) or completion-time retention (*B* =  − 0.074, 95% CI [− 0.332, 0.183], *t*(26) =  − 0.59, *p* = 0.558, *R*^2^ = 0.013, *β* =  − 0.116). Rigid Perfectionism was likewise not associated with retention slopes for path length (*B* =  − 3.76, 95% CI [− 21.40, 13.89], *t*(26) =  − 0.44, *p* = 0.665, *R*^2^ = 0.007, *β* =  − 0.085) or completion time (*B* = 0.138, 95% CI [− 0.227, 0.504], *t*(26) = 0.78, *p* = 0.443, *R*^2^ = 0.023, *β* = 0.151). The Condition by Self-Critical Perfectionism interaction did not reach statistical significance, *F*(1, 51) = 0.42, *p* = 0.521, partial *η*^2^ = 0.008. We found no main effect of Condition, *F*(1, 51) = 0.12, *p* = 0.728, partial *η*^2^ = 0.002, or Self-Critical Perfectionism, *F*(1, 51) = 2.37, *p* = 0.130, partial *η*^2^ = 0.044 (Fig. 2).

## Discussion

This study examined whether increasing working memory demands during practice is associated with differences in how perfectionistic concerns relate to the acquisition and retention of laparoscopic skills. Novice participants practiced a basic simulator task, and retention was assessed on a separate day. Performance was measured by completion time and total instrument path length.

We found no evidence that perfectionistic strivings were related to retention in either condition. For perfectionistic concerns, the pattern differed across conditions. In the control condition, this trait was associated with poorer retention of movement efficiency, while completion time was unaffected. This aligns with our previous work [15], suggesting that this type of negative self-evaluation can hinder the development and retention of efficient instrument movements during early MIS skill acquisition. In the working-memory load condition, no association was observed between perfectionistic concerns and retention at follow-up.

Taken together, these results are consistent with the possibility that increasing cognitive demands during practice may weaken the link between perfectionistic concerns and reduced retention of movement efficiency, although this interpretation should be confirmed in larger trainee samples.

### Implications

Our findings are consistent with the hypothesis that increasing working memory demands may weaken the link between perfectionistic concerns and poorer retention, potentially by reducing self-critical rumination and anxiety. Individuals high in perfectionistic concerns tend to fixate on shortcomings when performance falls short, which can fuel rumination and interfere with task-focused problem solving [25]. Perfectionistic concerns are also associated with anxiety about discrepancies between one’s performance and perceived expectations [27]. In this context, a concurrent working memory demand may limit the cognitive capacity available for self-evaluative thoughts, supporting sustained attention to task execution.

At the same time, although prior research links perfectionism to rumination [31] and suggests that increased working memory demands can reduce negative self-focused thinking [34], we did not measure state rumination, anxiety, or attentional focus during practice. Because testing mediating mechanisms was outside the scope of the present study, alternative explanations for the observed pattern remain plausible.

Although perfectionistic strivings are associated with higher task investment [17, 20], this did not lead to higher learning gains in this study, which is in line with our previous work [15]. One possible explanation is that the processes underlying perfectionistic strivings influence skill progression indirectly through sustained effort and the continuous raising of performance standards over time, rather than directly affecting immediate performance. When considering the effort invested to reach a given level of performance, it is useful to distinguish between absolute and relative performance. Absolute performance refers to the final level of skill or accuracy attained, whereas relative performance takes into account how efficiently this level is reached (e.g., per unit of practice time, cognitive effort, or emotional cost). Internally oriented perfectionists may reach higher absolute performance levels over time, but may do so less efficiently during the learning process than individuals who are less demanding of their own performance [41, 42].

The results of this study contribute to our understanding of how the effects of perfectionistic tendencies may be accommodated in surgical education. While group-level learning and retention were not improved, the pattern suggests that increasing cognitive demands during practice may be a feasible training constraint for trainees who struggle with self-critical focus. This supports the idea that tailoring training conditions to psychological barriers could help optimize learning for some learners.

Reduced self-critical rumination may also make practice feel less aversive and more engaging, which could benefit motivation and well-being over time. Future studies should test these potential emotional and motivational effects directly.

Although we used the BTPS to operationalize perfectionistic traits for research purposes, the present findings do not support using this or other personality questionnaires as a routine screening tool in skills training. Given that perfectionistic concerns are common in surgical training, it may be more feasible to offer optional training tools such as the working memory manipulation used here to trainees who self-identify as perfectionistic or who notice persistent worry and self-critical monitoring during practice.

### Limitations

This study has limitations. Participants were young and laparoscopically naïve, and training occurred in a simulated, low-stakes setting with minimal evaluative pressure. Effects of the working-memory manipulation may differ in surgical trainees working under higher stakes, time pressure, fatigue, and formal assessment. Our findings should therefore be viewed as proof-of-concept for early skill acquisition and replicated in trainees and clinically embedded, higher-stakes training contexts.

We used a standard visuomotor manipulation intended to increase reliance on visuospatial working memory. However, because we did not include a workload assessment, the extent to which it selectively taxed working memory was inferred from the task design rather than directly quantified.

Because the present study used a modest sample size, definitive conclusions about mitigating effects would require formal interaction testing in a larger, adequately powered sample. Future studies designed and powered specifically to detect moderation would therefore be valuable.

## Future research directions

Future studies could test the proposed mechanism with greater precision by measuring state rumination and anxiety during practice, alongside indicators of attentional allocation and strategy use such as self-report focus ratings or gaze and other behavioural markers. This would help determine whether any benefit is driven by reduced negative self-focus, changes in strategy, increased automaticity, or a combination of these factors.

Replication in authentic training environments, such as residency skills labs or supervised operating room training, and under stronger evaluative pressure would further establish the robustness and practical relevance of the findings.

Retention was assessed after 48 h, so the durability of the observed effects beyond this interval remains unknown. Future studies should include longer follow-up periods to evaluate longer-term retention and transfer.

Future work should also examine whether similar effects occur when working memory is taxed in other ways, to assess the generalizability of this approach. Finally, studies could test whether this manipulation influences emotional outcomes, such as enjoyment and motivation to practice, particularly among trainees with perfectionistic tendencies.

## Conclusion

This study adds to our understanding of how increasing cognitive demands during practice may relate to perfectionistic concerns in surgical skill learning. At the group level, taxing working memory during practice was feasible and did not impair acquisition or retention. The observed pattern of condition-specific associations suggests that the relationship between perfectionistic concenrs and reduced retention of movement efficiency may be weaker under a working-memory demanding training context. These findings motivate further, adequately powered studies to test whether such training constraints can help reduce self-critical interference and support learning in trainees.
